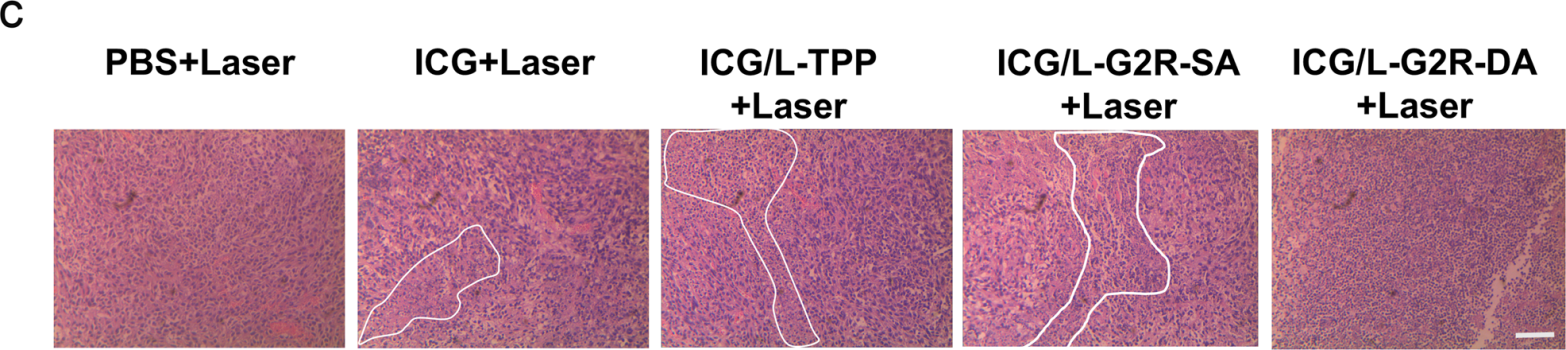# Author Correction: Mitochondrion-specific dendritic lipopeptide liposomes for targeted sub-cellular delivery

**DOI:** 10.1038/s41467-025-61381-1

**Published:** 2025-06-26

**Authors:** Lei Jiang, Sensen Zhou, Xiaoke Zhang, Cheng Li, Shilu Ji, Hui Mao, Xiqun Jiang

**Affiliations:** 1 MOE Key Laboratory of High Performance Polymer Materials and Technology, and Department of Polymer Science and Engineering, College of Chemistry and Chemical Engineering, Nanjing University, Nanjing, China; 2 Department of Radiology and Imaging Sciences, Emory University, Atlanta, GA USA

Correction to: *Nature Communications* 10.1038/s41467-021-22594-2, published online 22 April 2021

In the version of the article initially published, in Fig. 7c, the “ICG/L-G2R-SA+Laser” image was incorrect and has now been amended in the HTML and PDF versions of the article, as seen in Fig. 1.

Fig. 1 | Original Fig. 7c
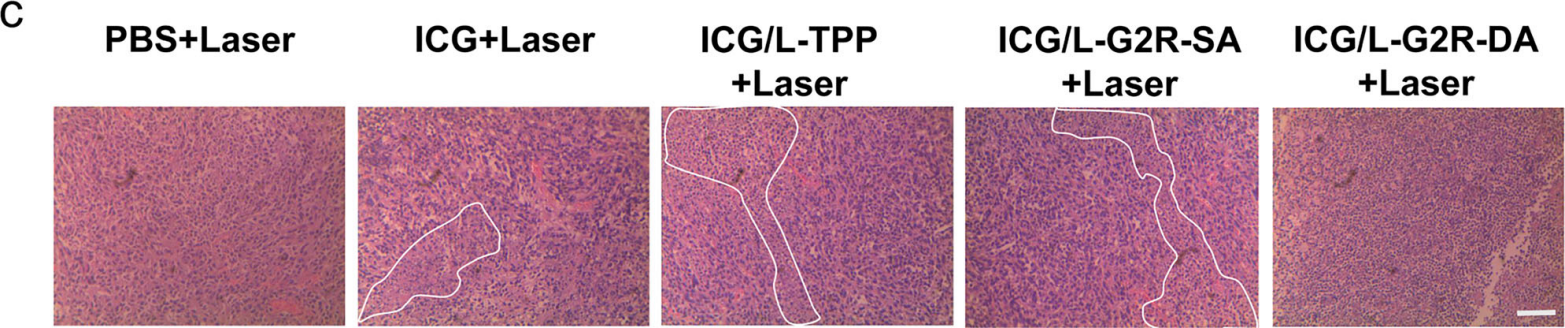


Fig. 1 | corrected Fig. 7c