# Supplementary material for: Mitochondrion-specific dendritic lipopeptide liposomes for targeted sub-cellular delivery
Source: Nat Commun. 2021 Apr 22;12:2390. doi: 10.1038/s41467-021-22594-2 (PMC8062597; doi:10.1038/s41467-021-22594-2)
Supplement: Supplementary file 1 — Supplementary Information [file 41467_2021_22594_MOESM1_ESM.pdf]

# Supporting Information

# Mitochondrion-Specific Dendritic Lipopeptide Liposomes for Targeted Sub-Cellular Delivery

Lei Jiang[1], Sensen Zhou[1], Xiaoke Zhang[1], Cheng Li[1], Shilu Ji[1], Hui Mao[2] and Xiqun Jiang[1*]

[1]MOE Key Laboratory of High Performance Polymer Materials and Technology, and Department of Polymer Science & Engineering, College of Chemistry & Chemical Engineering, Nanjing University, Nanjing 210093, China

[2]Department of Radiology and Imaging Sciences, Emory University, Atlanta, Georgia 30329, USA.

* Corresponding author (email: jiangx@nju.edu.cn)

**1 Experimental Method and Supplementary Scheme**

**2 Supplementary Results, Table and Figures**

## 1 Experimental Section

### 1.1 Materials and Methods

**Materials**

H-Lys-OMe·2HCl, Boc-Lys(Boc)-OH, Boc-Arg(Pbf)-OH, 1-hydroxybenzotriazole hydrate (HOBT) and 2-($^1$H-benzotriazole-1-yl)-1,1,3,3-tetramethyluronium hexafluorophosphate (HBTU) were purchased from GL Biochem. Ltd. (Shanghai, China). *N, N*-diisopropylethylamine (DIPEA) and trifluoroacetic acid (TFA) were purchased from J&K Scientific Company (Beijing, China). Indocyanine Green (ICG) was purchased from Aladdin Company (Shanghai, China). 2,3-Dimethylmaleic anhydride (DA) and succinic anhydride (SA) were purchased from Sigma Company (St Louis, USA). Soyabeanphosphatidylcholine (SPC) was purchased from Lipoid GmbH (Ludwigshafen, Germany). 1, 2-Distearoyl-sn-glycero-3-phosphoethanolamine (DSPE) and DSPE-PEG$_{2000}$ were purchased from CordenPharma Company (Swizerland). Mitotracker Green FM was purchased from KeyGEN Company (Nanjing, China). All other solvents were purchased from Tianjin Kemiou Chemical Reagent Company (Tianjin, China). Roswell Park Memorial Institute (RPMI)-1640 medium, penicillin, streptomycin and fetal bovine serum (FBS) were purchased from Hyclone (USA). 4T1 cell line (mouse breast cancer cell) was purchased from Chinese Academy of Science Cell Bank for Type Culture Collection (Shanghai, China). BALB/c mice (18 ± 2 g, 5-6 weeks old) were purchased from Qinglongshan Laboratory Animal Center (Nanjing, China).

**Methods**

### 1.2 Synthesis of dendritic lipopeptides

#### 1.2.1 1, 2-Distearoyl-sn-glycero-3-phosphoethanolamine-G1 (argine) (G1R)

DSPE (0.75 g, 1.00 mmol) was dissolved in anhydrous trichloromethane (15 mL) under nitrogen atmosphere. DIPEA (1.00 mL, 6.00 mmol) was added to the solution in an ice bath. Boc-Arg (Pbf)-OH (0.79 g, 1.5 mmol), HOBT (0.2 g, 1.5 mmol) and HBTU (0.57 g, 1.5 mmol) dissolved in anhydrous DMF (2.5 mL) was added into the reaction flask containing DSPE. The solution was stirred under nitrogen in an ice bath for 30 min and at room temperature for another 24 h. The mixture was washed with saturated

NaHCO$_3$, NaHSO$_4$, and NaCl solution for several times. The mixture was dried with MgSO$_4$ for 2 h. After the removal of solvents, the mixture was purified by silica gel column chromatography (DCM/MeOH, 12/1, V/V) to obtain **Compound 1** (yield: 80%).

**Compound 1** was dried in a vacuum and dissolved in anhydrous dichloromethane (DCM)/TFA (1:1, 10 mL) for 4 hours to put off tert-butyl groups. The mixture was concentrated, and the product was treated with anhydrous diethyl ether to obtain **Compound 2** (yield: 90%) (Supplementary Scheme 1). $^1$H NMR (400 MHz, CDCl$_3$) δ 7.52 (m, 2H), 5.30 (s, 1H), 3.93-4.62 (m, 8H), 3.45-3.5 1 (m, 3H), 2.19-2.27 (t, 4H), 1.92 (s, 2H), 1.34-1.57 (m, 8H), 1.25-1.30 (m, 56H), 0.86-0.89 (t, 6H).

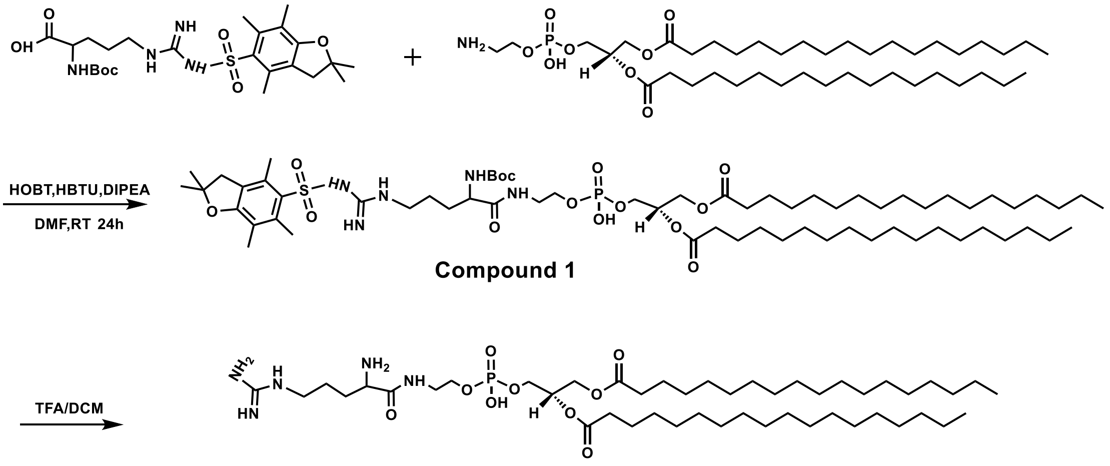


**Supplementary Scheme 1.** Synthetic route of dendritic lipopeptides G1R.

## 1.2.2 Synthesis of 1, 2-Distearoyl-sn-glycero-3-phosphoethanolamine-G2 (argine-lysine) (G2R)

H-Lys-OMe.2HCl (2.00 g, 8.6 mmol), Boc-Arg(Pbf)-OH (13.56 g, 25.8 mmol), HBTU (9.78 g, 25.8 mmol) and HOBT (3.36 g, 25.8 mmol) were dissolved in anhydrous DMF (50 mL) in nitrogen atmosphere. DIPEA (11.4 mL, 68.8 mmol) was added in the ice-water bath. This reaction mixture was stirred at room temperature for 2 days. Then, the mixture was washed with saturated NaHCO$_3$, NaHSO$_4$, and NaCl solution for several times. The mixture was dried with MgSO$_4$ for 2 h. After the removal

of solvents, the mixture was purified by silica gel column chromatography (DCM/MeOH, 12/1, V/V) to obtain **Compound 3** (yield: 86%).

**Compound 3** (8.00 g, 6.8 mmol) was treated with NaOH in 100 mL MeOH (1 mol/L) for 4 hours to expose carboxyl groups. After the removal of MeOH, the mixture was dissolved in $H_2O$ and adjusted to neutral pH value. **Compound 4** could be extracted by DCM and dried with $MgSO_4$ for 2 h (yield: 72%).

DSPE (1.5 g, 2.00 mmol) was dissolved in anhydrous trichloromethane (30 mL) under nitrogen atmosphere. DIPEA (2.00 mL, 12.00 mmol) was added to the solution under stirring at 0 °C. **Compound 4** (3.62 g, 3 mmol)**,** HOBT (0.4 g, 3 mmol) and HBTU (1.14 g, 4 mmol) dissolved in anhydrous DMF (5 mL) was added to the reaction flask. The solution was stirred under nitrogen in ice bath for 30 min and at room temperature for another 24 h. The mixture was washed with saturated $NaHCO_3$, $NaHSO_4$, and NaCl solution for several times. The mixture was dried with $MgSO_4$ for 2 hours. After the removal of solvents, the mixture was purified by silica gel column chromatography (DCM/MeOH, 12/1, V/V) to obtain **Compound 5** (yield: 80%).

**Compound 5** was dried in vacuum and dissolved in anhydrous dichloromethane (DCM)/TFA (1:1, 10 mL) for 4 hours to put off tert-butyl groups. The mixture was concentrated, and the product was treated with anhydrous diethyl ether to obtain **Compound 6** (yield: 90%) (Supplementary Scheme 2)**.** $^1H$ NMR (400 MHz, CDCl3) δ 6.48 (s, 1H), 5.18 (s, 1H), 4.26-4.40 (m, 2H), 3.88-4.16 (m, 4H), 3.60-3.72 (m, 2H), 2.86-2.98 (m, 6H), 1.98-2.52 (m, 12H), 1.44-1.55 (m, 8H), 1.25-1.33 (m, 58H), 0.86-0.89 (t, 6H).

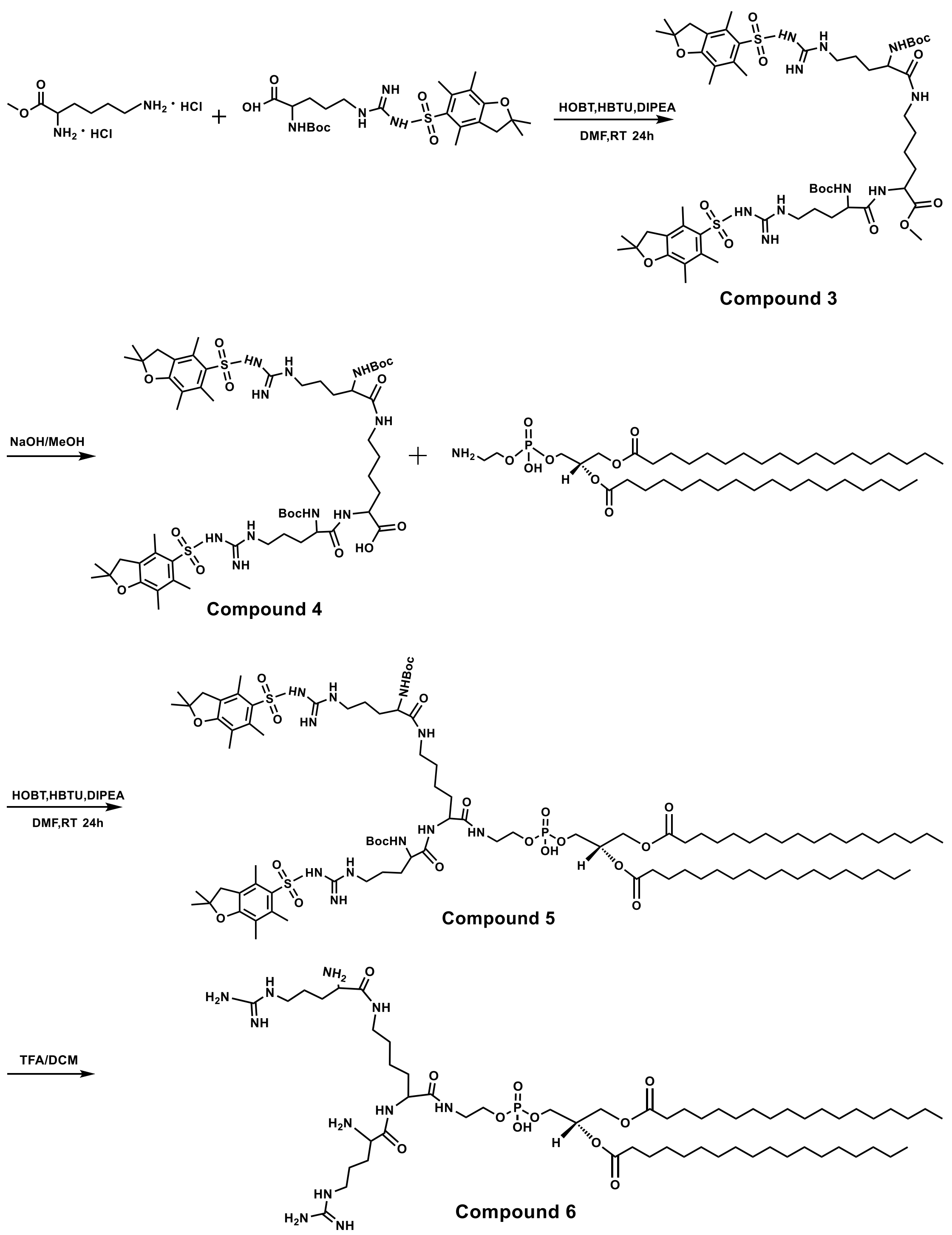

**Supplementary Scheme 2.** Synthetic route of dendritic lipopeptides G2R.

### 1.2.3 Synthesis of 1, 2-Distearoyl-sn-glycero-3-phosphoethanolamine-G2 (lysine-lysine) (G2K)

H-Lys-OMe.2HCl (1.00 g, 4.3 mmol), Boc-L-Lys(Boc)-OH (4.46 g, 12.9 mmol), HBTU (4.89 g, 12.9 mmol) and HOBT (1.68 g, 12.9 mmol) were dissolved in

anhydrous DMF (25 mL) in nitrogen atmosphere. DIPEA (5.7 mL, 34.4 mmol) was added in the ice-water bath. This reaction mixture was stirred at room temperature for 48 hours. Then, the mixed solution was washed with saturated $NaHCO_3$, $NaHSO_4$, and NaCl solution for several times. The mixture was dried with $MgSO_4$ for 2 h. After the removal of solvents, the mixture was purified by silica gel column chromatography (DCM/MeOH, 12/1, V/V) to obtain **Compound 7** (yield: 85%). **Compound 7** (4.00 g, 3.4 mmol) was treated with NaOH in 50 mL MeOH (1 mol/L) for 4 hours to expose carboxyl groups. After the removal of MeOH, the mixture was dissolved in $H_2O$ and adjusted to neutral pH value. **Compound 8** could be extracted by DCM and dried with $MgSO_4$ for 2 h (yield: 74%). DSPE (0.75 g, 1.00 mmol), **Compound 8** (1.81 g, 1.5 mmol), HOBT (0.27 g, 2 mmol) and HBTU (0.57 g, 2 mmol) were dissolved in anhydrous trichloromethane (25 mL) and anhydrous DMF (3 mL) under nitrogen atmosphere. DIPEA (2.00 mL, 14.00 mmol) was added to the above mixed solution under stirring at 0°C. The solution was stirred under nitrogen for another 48 h at room temperature. The mixture was washed with saturated $NaHCO_3$, $NaHSO_4$, and NaCl solution for several times. The mixture was dried with $MgSO_4$ for 2 hours. After the removal of solvents, the mixture was purified by silica gel column chromatography (DCM/MeOH, 12/1, V/V) to obtain **Compound 9** (yield: 81%). **Compound 9** was dried in vacuum and dissolved in anhydrous dichloromethane DCM/TFA (1:1, 10 mL) for 4 hours to put off tert-butyl groups. The mixture was concentrated, and the product was treated with anhydrous diethyl ether to obtain **Compound 10** (yield: 92%) (Supplementary Scheme 3). $^1$H NMR (400 MHz, D2O) δ 7,98 (d, 1H), 5,24 (t, 2H), 4.16-4.43 (m, 5H), 3.8-4.0 (m, 4H), 3.48-3.49 (t, 2H), 2,81-2.99 (m, 4H), 2.30-2.36 (m, 4H), 1.98-1.47 (m, 16H), 1.25-1.29 (m, 56H), 0.88 -0.90 (t, 6H).

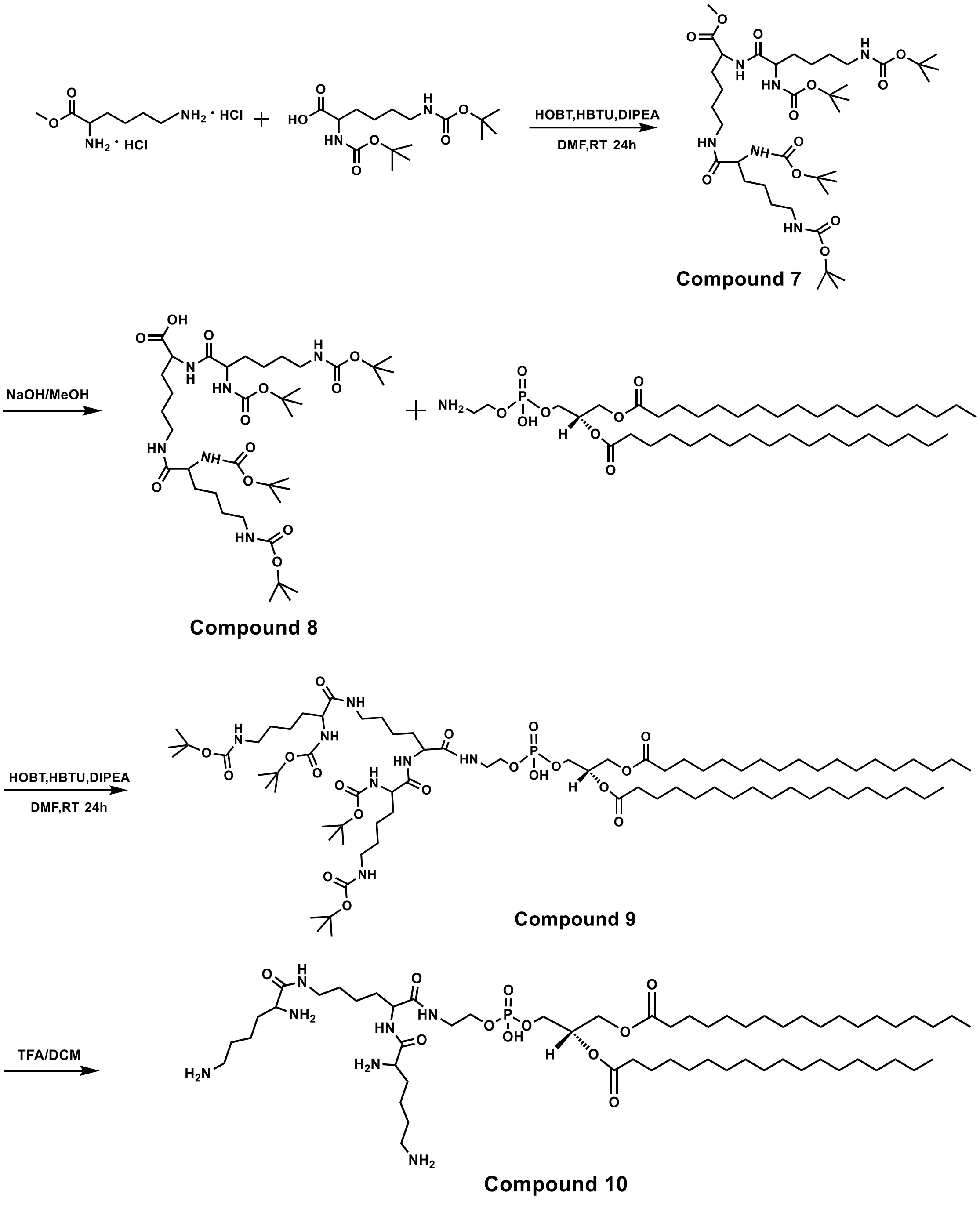


**Supplementary Scheme 3.** Synthetic route of G2K.

### 1.2.4 Synthesis of 1, 2-Distearoyl-sn-glycero-3-phosphoethanolamine-G2 (argine-lysine)-2,3-dimethylmaleic anhydride (G2R-DA)

**Compound 6** (1 g, 0.84 mmol) was dissolved in 10 mL of anhydrous dichloromethane. TEA (1.77 g, 2.86 mmol) and DA (2.2 g, 17.5 mmol) were added to the solution. The mixture was stirred under nitrogen in an ice bath for 30 min and at room temperature. After the solution was stirred at room temperature for 24 h, the

mixture consisting of TEA (1.77 g, 2.86 mmol) and DA (2.2 g, 17.5 mmol) was added. The solution was stirred at room temperature for an additional 24 hours. Then, the solution was added dropwise to an ice anhydrous diethyl ether (100 mL) and the precipitate appeared. The precipitate was dissolved in MeOH and dialyzed in a dialysis bag (Spectra/Por MWCO = 1000) against fresh deionized water (pH 7.4) for three days at 4 °C. The outer phase was replaced with fresh deionized water (pH 7.4) every 4 hours. The solution in the bag was freeze-dried to received **Compound 11** (yield: 61.1%) (Supplementary Scheme 4). $^1$H NMR (400 MHz, $CDCl_3$) δ 11.84 (s, 1H), 5.19 (s, 1H), 5.33 (s, 1H), 4.28-4.44 (m, 1H), 3.77-4.22 (m, 6H), 3.64 (t, 2H), 3.47-3.49 (m, 4H), 2.93 (t, 2H), 2.53-2.62 (m, 4H), 2.12-2.28 (m, 4H), 1,94 (s, 24H), 1.46-1.58 (m, 8H), 1.25-1.36 (m, 56H), 0.86-0.89 (t, 6H).

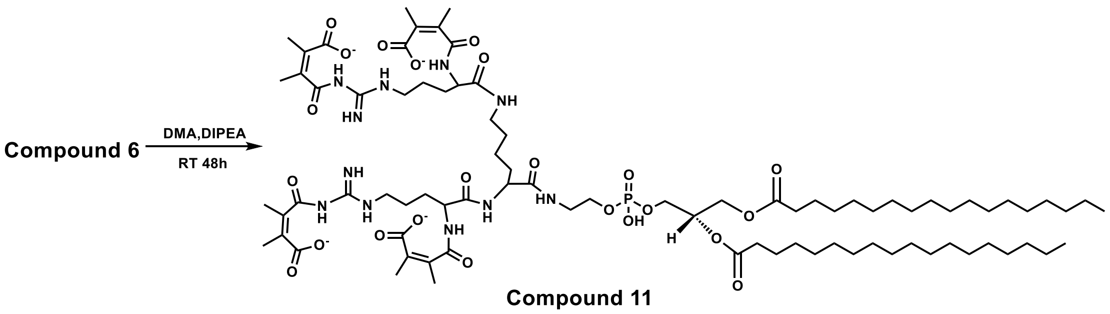


**Supplementary Scheme 4.** Synthetic route of G2R-DA.

### 1.2.5 Synthesis of 1, 2-Distearoyl-sn-glycero-3-phosphoethanolamine-3-carboxypropyl triphenyl-phosphonium bromide (DTPP)

DSPE (0.1 g, 0.133 mmol) was dissolved in anhydrous trichloromethane (15 mL) under nitrogen atmosphere. DIPEA (0.25 mL, 1.3 mmol) was added to the solution under stirring at 0 °C. 3-carboxypropyl triphenyl-phosphonium bromide, (3-CTPP) (0.084 g, 0.19 mmol), HOBT (0.025 g, 0.19 mmol) and HBTU (0.071 g, 0.19 mmol) dissolved in anhydrous DMF (2.5 mL) and added to the reaction flask containing DSPE. The solution was stirred under nitrogen in ice bath for 30 mins and at room temperature for another 24 hours. The mixture was washed with saturated $NaHCO_3$, HCl, and NaCl solution for several times. The mixture was dried with $MgSO_4$ for 2 hours. After the

removal of solvents, the mixture was purified by silica gel column chromatography (DCM/MeOH, 12/1, V/V) to obtain **Compound 12** (yield: 72%) (Supplementary Scheme 5). $^1$H NMR (400 MHz, $CDCl_3$) δ 8.66 (s, 2H), 7.68-7.86 (m, 15H), 5.18 (s, 1H), 4.09-4.35 (m, 4H), 3.53-3.81 (m, 2H), 2.72-2.96 (m, 3H), 2.28-2.33 (m, 4H), 1.93 (t, 2H), 1.6 (t, 2H), 1.23-1.25 (m, 56H), 0.86-0.89 (t, 6H).

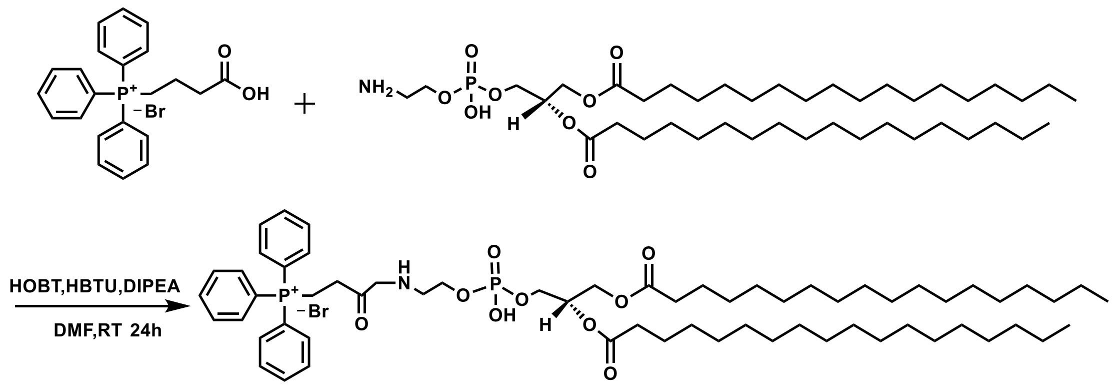


Compound 12

**Supplementary Scheme 5.** Synthetic route of DTPP.

### 1.2.6 Synthesis of The control compounds 1, 2-Distearoyl-sn-glycero-3-phosphoethanolamine-G2 (argine-lysine)-succinic anhydride (G2R-SA)

**Compound 6** (0.5 g, 0.42 mmol), succinic anhydride (SA) (1.0 g, 9.7 mmol) and TEA (0.3 g, 2.5 mmol) were dissolved in DMF (5 mL). The solution was added dropwise to ice anhydrous diethyl ether (100 mL) and the precipitate appeared. The precipitate was dissolved in deionized water and dialyzed in a dialysis bag (Spectra/Por MWCO = 1000) for three days. The outer phase was replaced with fresh deionized water every 4 hours. The solution in the bag was freeze-dried to afford **Compound 13** (yield: 71.1%) (Supplementary Scheme 6). $^1$H NMR (400 MHz, $CDCl_3$) δ 7.83 (t, 2H), 7.0 (t, 1H), 5.33 (s, 1H), 4.59 (s, 1H), 4.33-4.36 (d, 2H), 4.12-4.15 (d, 2H), 3.71-3.91 (m, 4H), 3.63-3.68 (m, 3H), 3.29-3.31 (m, 4H), 2.58-3.12 (m, 8H), 2.49-2.57 (t, 4H), 2.27-2.29 (d, 4H), 2.08 (s, 1H), 1.93 (s, 1H), 1.36-1.56 (m, 4H), 1.35-1.41 (m, 4H), 1.19-1.25 (m, 56H), 1.03-1.07 (m, 2H), 0.86-0.89 (t, 6H).

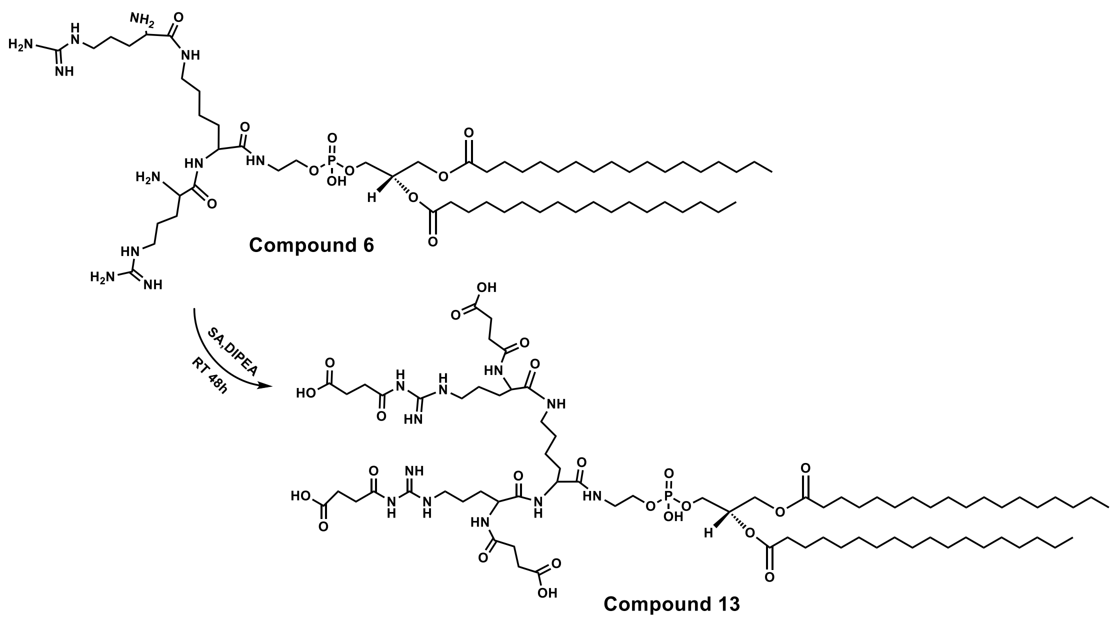


**Supplementary Scheme 6.** Synthetic route of dendritic lipopeptides G2R-SA.

### 1.2.7 Synthesis of 1, 2-Distearoyl-sn-glycero-3-phosphoethanolamine-G2 (lysine-lysine) (G2K)-2,3-dimethylmaleic anhydride (G2R-DA)

**Compound 10** (1 g, 0.88 mmol) was dissolved in 10 mL of anhydrous dichloromethane. TEA (1.77 g, 2.86 mmol) and DA (2.2 g, 17.5 mmol) were added to the solution. The mixture was stirred under nitrogen in an ice bath for 30 min and at room temperature. After the solution was stirred at room temperature for 24 h, the mixture consisting of TEA (1.77 g, 2.86 mmol) and DA (2.2 g, 17.5 mmol) was added. The solution was stirred at room temperature for an additional 24 hours. Then, the solution was added dropwise to an ice anhydrous diethyl ether (100 mL) and the precipitate appeared. The precipitate was dissolved in MeOH and dialyzed in a dialysis bag (Spectra/Por MWCO = 1000) against fresh deionized water (pH 7.4) for three days at 4 °C. The outer phase was replaced with fresh deionized water (pH 7.4) every 4 hours. The solution in the bag was freeze-dried to received **Compound 14** (yield: 60.5%) (Supplementary Scheme 7). $^{1}$H NMR (400 MHz, $CDCl_3$) δ 11.3 (s, 1H), 8.1 (s, 1H), 5.22 (s, 1H), 4.36 (m, 2H), 4.14 (m, 2H), 3.92 (m, 4H), 3.65-3.68 (m, 2H), 3.45-3.51 (m, 2H), 3.07-3.12 (m, 6H), 2.28 (t, 4H), 2.03-1.94 (m, 12H), 1.68 (s, 24H), 1.18-1.25 (m, 56H), 0.86-0.9 (t, 6H).

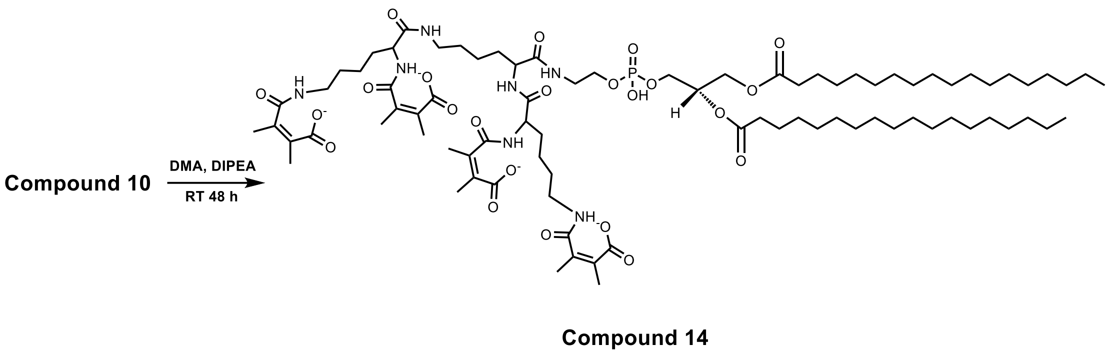


**Supplementary Scheme 7.** Synthetic route of dendritic lipopeptides G2K-DA.

### 1.3 Improved efficacy was resulted from the delivery of ICG to tumor cell mitochondria

Two types of liposomes, a charge-reversible L-G2R-DA and non-charge-reversible L-G2R-SA were used to carry ICG. Firstly, ICG-loaded liposome ICG/L-G2R-SA and ICG/L-G2R-DA were incubated with 4T1 cells at pH 7.4 at 37 °C for 2 h, and then collected cell pellets were washed twice with cold PBS and the cells were centrifuged to collection. ICG from the cells were extracted using methanol. Mitochondrial isolation was carried out using a cell mitochondria isolation kit and ICG from the mitochondria were extracted using methanol. The concentrations of ICG were measured using fluorescent spectrometer. Subsequently, we investigate cell apoptosis of ICG/L-G2R-SA or ICG/L-G2R-DA-internalized 4T1 cells upon NIR irradiation. The cells were irradiated by 808 nm laser for 1 min (0.65 W/cm$^2$) and incubated for an additional 12 h. After washing and centrifugation, the Annexin V-FITC/PI Apoptosis Detection (KeyGEN, Nanjing, China) was employed by flow cytometry in accordance with the specification of kit. Next, we investigated cytotoxicity of ICG/L-G2R-SA or ICG/L-G2R-DA- internalized 4T1 cells upon NIR irradiation. The cells were irradiated by 808 nm laser for 3 min (0.65 W/cm$^2$) and incubated for an additional 12 h. After washing and centrifugation, the propidium iodide (PI) and calcein AM Live/Dead Detection (KeyGEN, Nanjing, China) were employed for fluorescence spectrometer measurement and observation in accordance with the specification of kit.

## 2 Results

### 2.1 Characterizations of dendritic lipopeptides

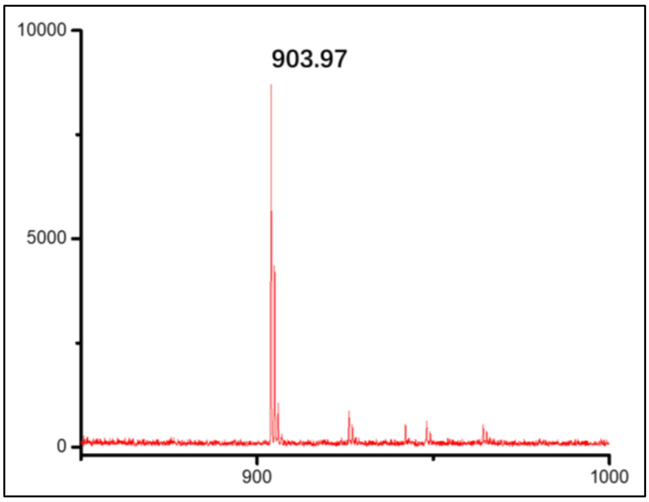


**Supplementary Figure 1.** MALDI-TOF MS spectrum of Compound 2 G1R.

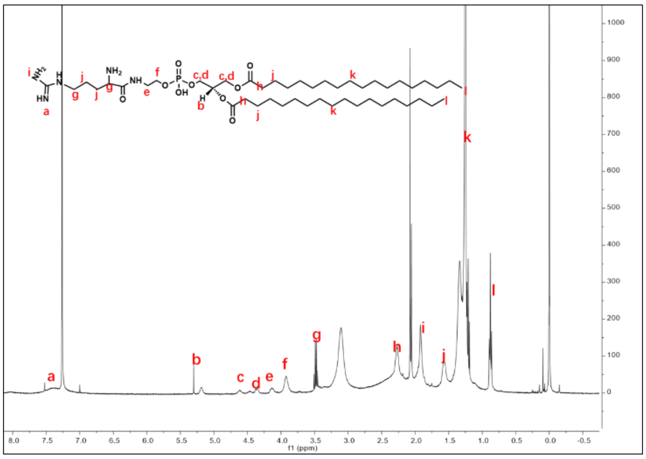

**Supplementary Figure 2.** $^1H$-NMR spectrum of Compound 2 G1R in $CDCl_3$.

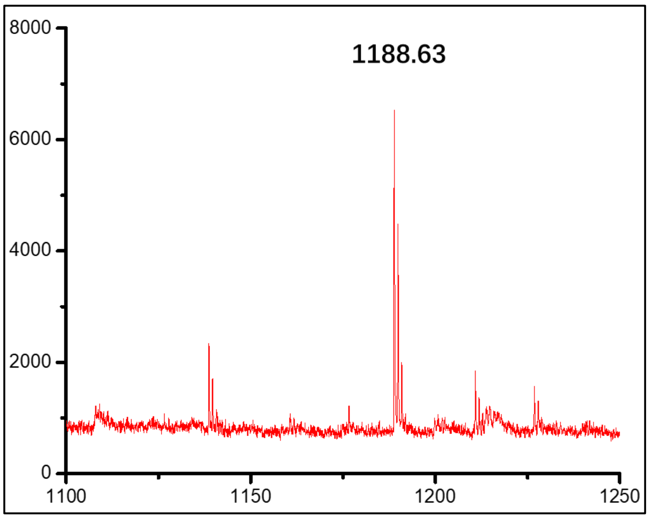


**Supplementary Figure 3.** MALDI-TOF MS spectrum of Compound 6 G2R.

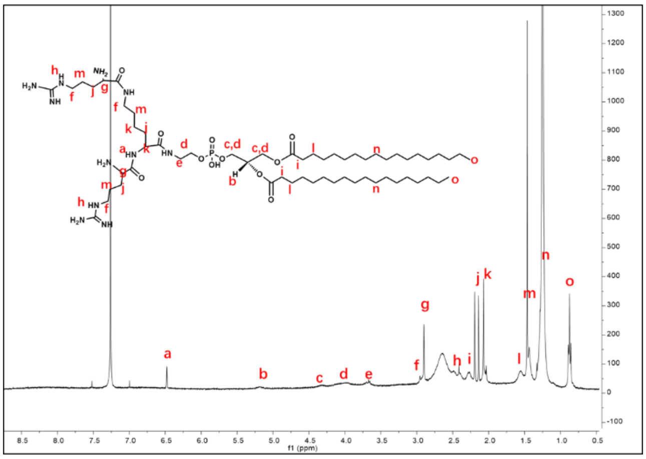

**Supplementary Figure 4.** $^{1}$H-NMR spectrum of Compound 6 G2R in $CDCl_3$.

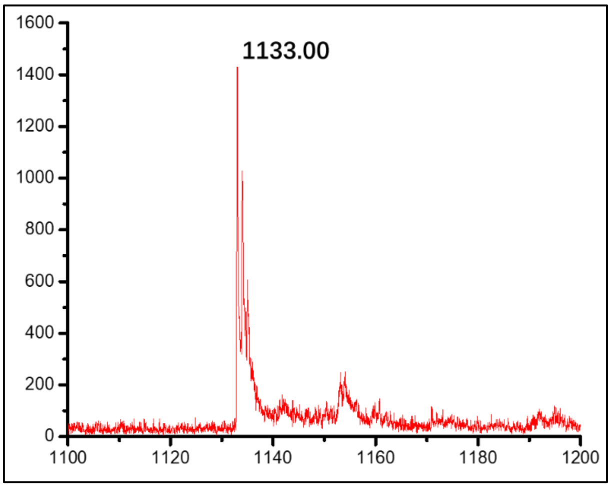


**Supplementary Figure 5.** MALDI-TOF MS spectrum of Compound 10 G2K.

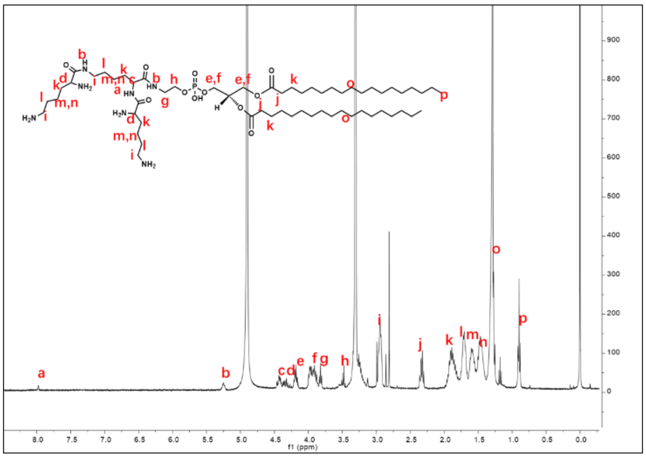

**Supplementary Figure 6.** $^{1}$H-NMR spectrum of Compound 10 G2K in D2O.

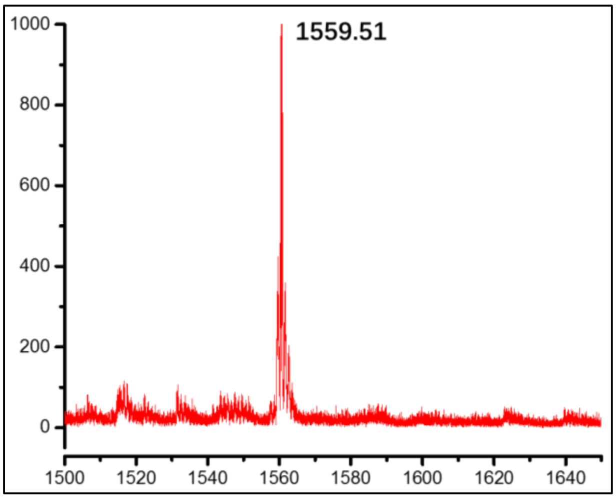


**Supplementary Figure 7.** MALDI-TOF MS spectrum of Compound 11 G2R-DA.

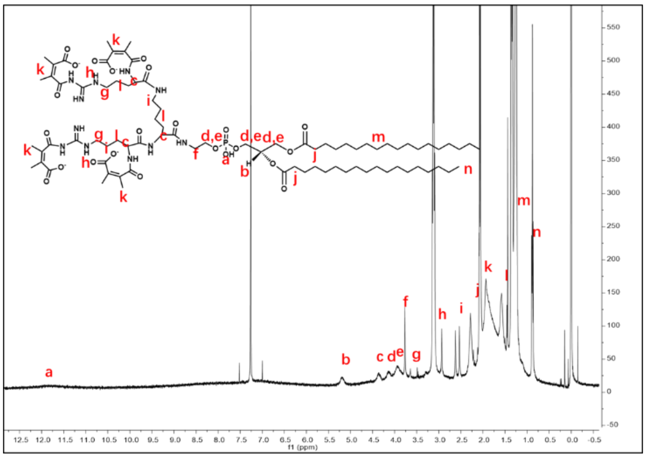

**Supplementary Figure 8.** $^1$H-NMR spectrum of Compound 11 G2R-DA in $CDCl_3$.

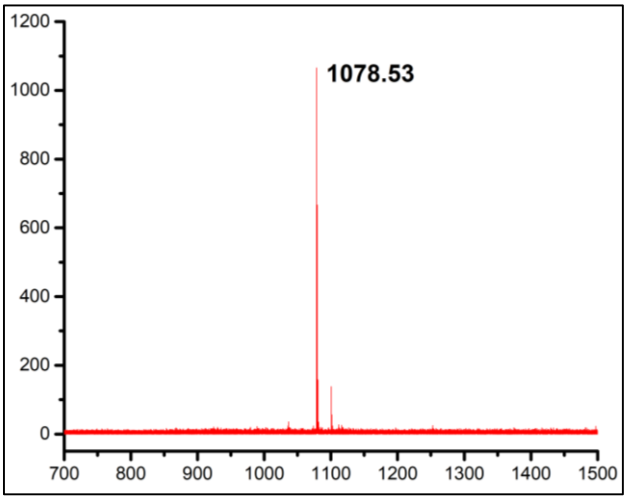


**Supplementary Figure 9.** MALDI-TOF MS spectrum of Compound 12 DTPP.

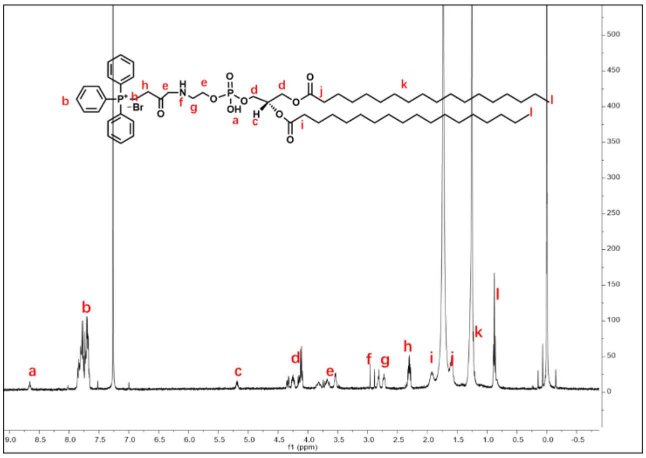


**Supplementary Figure 10.** $^{1}$H-NMR spectrum of Compound 12 DTPP in $CDCl_3$.

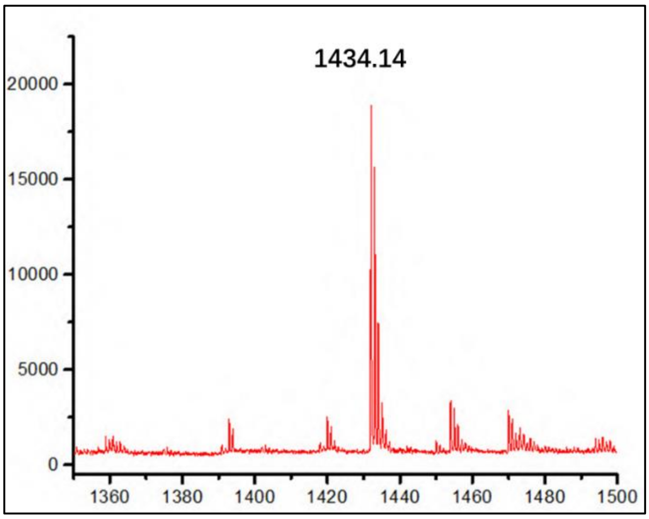


**Supplementary Figure 11.** MALDI-TOF MS spectrum of Compound 13 G2R-SA.

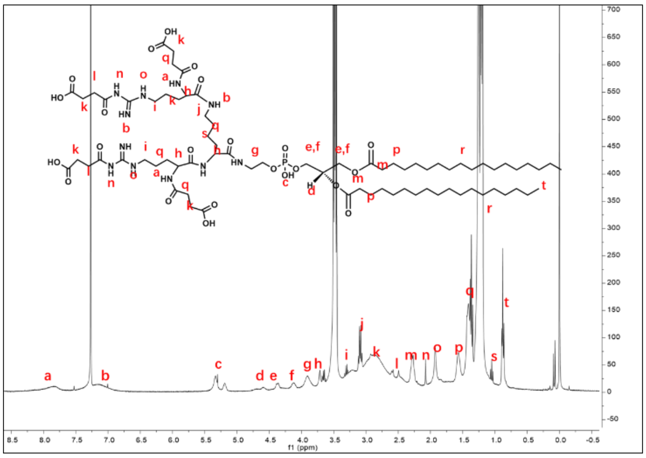

**Supplementary Figure 12.** $^{1}$H-NMR spectrum of Compound 13 G2R-SA in $CDCl_3$.

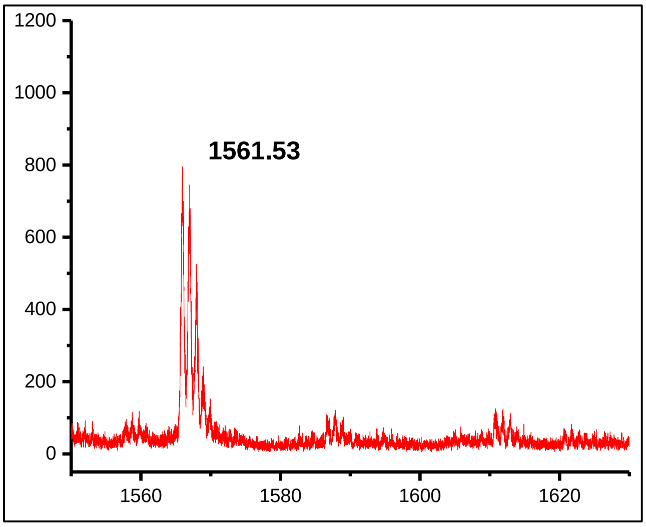


**Supplementary Figure 13.** MALDI-TOF MS spectrum of Compound 14 G2K-DA.

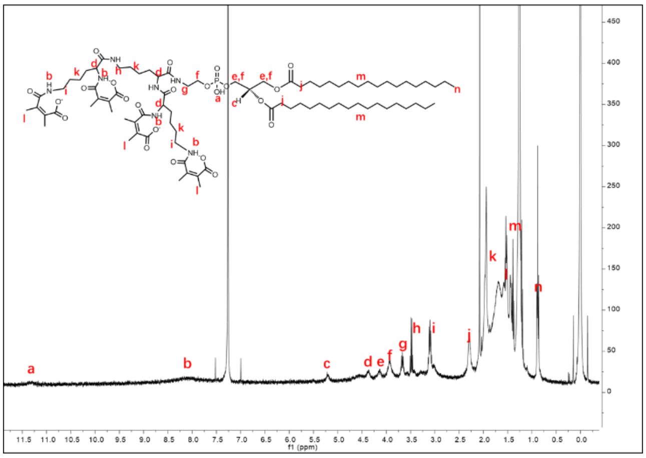

**Supplementary Figure 14.** $^{1}$H-NMR spectrum of Compound 14 G2K-DA in $CDCl_3$.

## 2.2 Characterizations of liposomes

**Table S1 Characterization of liposomes** (*n* = 3 independent samples, mean ± SEM).

| Name | Formulation ( mol:mol) | Size (nm) | PDI | Zeta | EE (%) |
|---|---|---|---|---|---|
| L-G2K-DA | S:C:P:K=5:1:0.35:1.2 | 104.0±2.5 | 0.28±0.01 | -13.48±0.74 | -- |
| ICG/L-TPP | S:C:P:T:I=5:1:0.35:2.4:1.3 | 139.4±2.5 | 0.24±0.02 | 23.67±0.12 | 97.29±0.72 |
| ICG/L-G2R-SA | S:C:P:Sa:I =5:1:0.35:1.2:1.3 | 108.4±1.6 | 0.25±0.01 | -14.88±0.61 | 96.62±0.45 |
| ICG/L-G2R-DA | S:C:P:D:I=5:1:0.35:1.2:1.3 | 106.7±1.8 | 0.23±0.01 | -15.02±1.12 | 97.68±1.07 |

Note: S, SPC. C, cholesterol. P, DSPE-$PEG_{2000}$. K, G2K-DA. T, DTPP. Sa, G2R-SA. D, G2R-DA. I, ICG. PDI, polydispersity index.

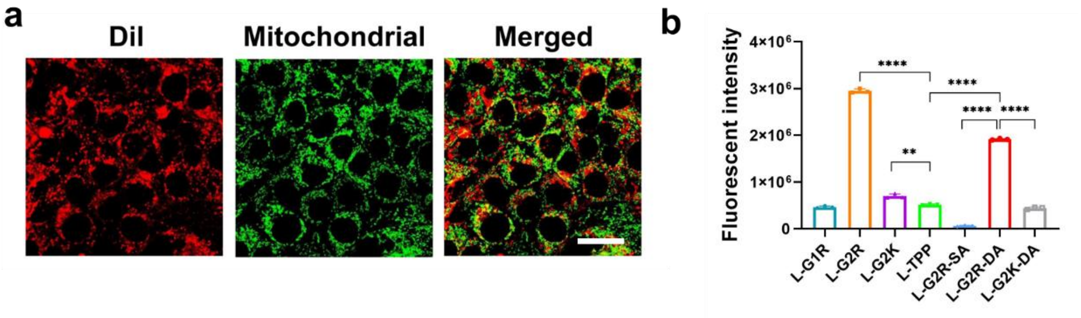


**Supplementary Figure 15. a** CLSM images showing mitochondrial localization of L-G2K-DA. 4T1 breast cancer cells were incubated with Dil-loaded liposomes for 12 h and then stained with Mitotracker Green FM. Areas with yellow fluorescence in the merged CLSM images denote the co-localization of the liposomes within mitochondria. The scale bar is 40 μm. **b** Flow cytometry analysis of accumulation of Dil-loaded liposomes in mitochondria of 4T1 cells. Data are presented as means ± SEM. ($n$ = 3 biologically independent samples). $P$ values were calculated by the two-tailed Student's $t$-test (**$p$=0.0031, ****$p < 0.0001$).

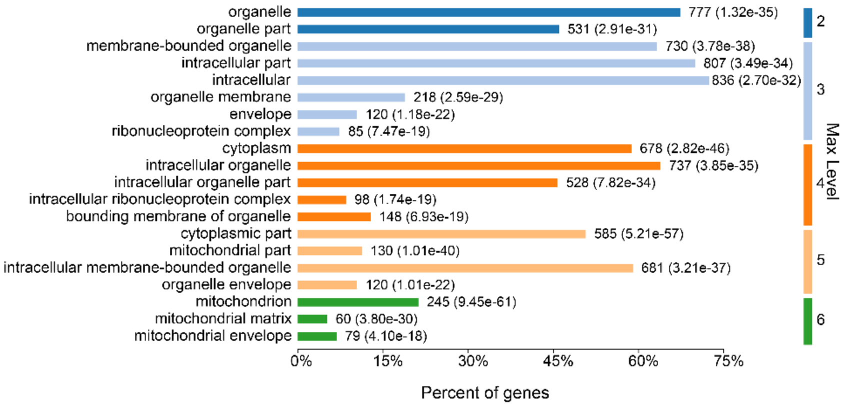


**Supplementary Figure 16**. The GeneOntology (GO) pathway analysis according to Cellular Component (CC) for the unique proteins bound on control group, liposomes without G2R ingredient.

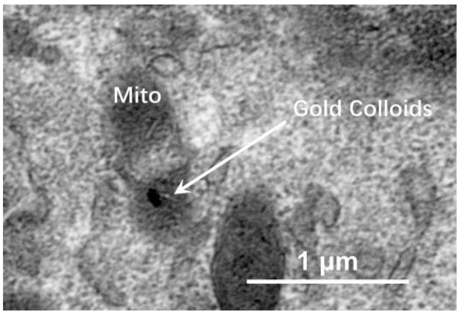


**Supplementary Figure 17**. TEM observation of mitochondrial delivery in living cells. ICG/L-G2R encapsulating Gold Colloid (10 nm in diameter) as a model macromolecule. Mito indicates mitochondria. Scale bar is 1 μm. Experiments were repeated three times independently.

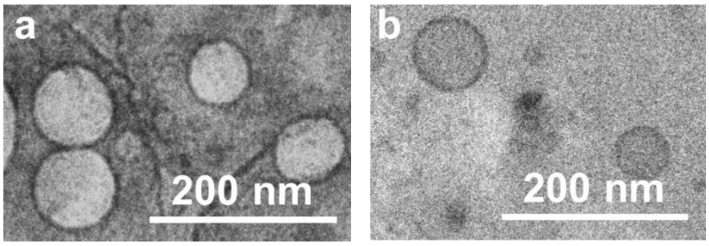


**Supplementary Figure 18.** Cyro-TEM images of **a** ICG/L-TPP, **b** ICG/L-G2R-SA. Scale bars are 200 nm. Experiments were repeated three times independently.

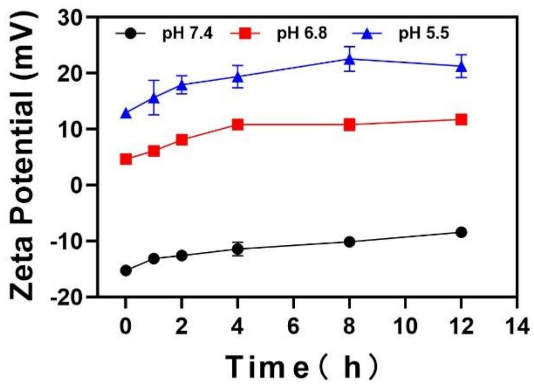


**Supplementary Figure 19.** Zeta potential of ICG/L-G2R-DA at different pH values within 12 h. Data are presented as means ± SEM. ($n$ = 3 independent samples).

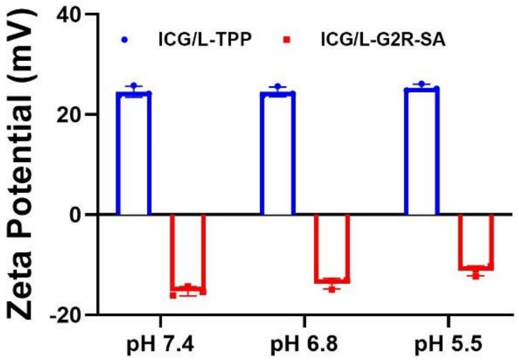


**Supplementary Figure 20.** Zeta potential of ICG/L-TPP and ICG/L-G2R-SA at different pH values at 12 h. Data are presented as means ± SEM. ($n$ = 3 independent samples).

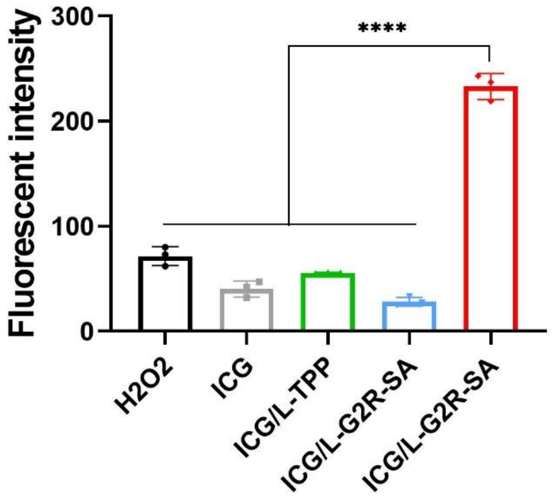


**Supplementary Figure 21.** ROS generation following incubation of the cells with PBS, $H_2O_2$, various ICG formulation and 808 nm laser irradiation (0.65 W/cm$^2$, 3 min). Data are presented as means ± SEM. ($n$ = 3 biologically independent samples). $P$ values were calculated by the two-tailed Student's $t$-test (****$p < 0.0001$).

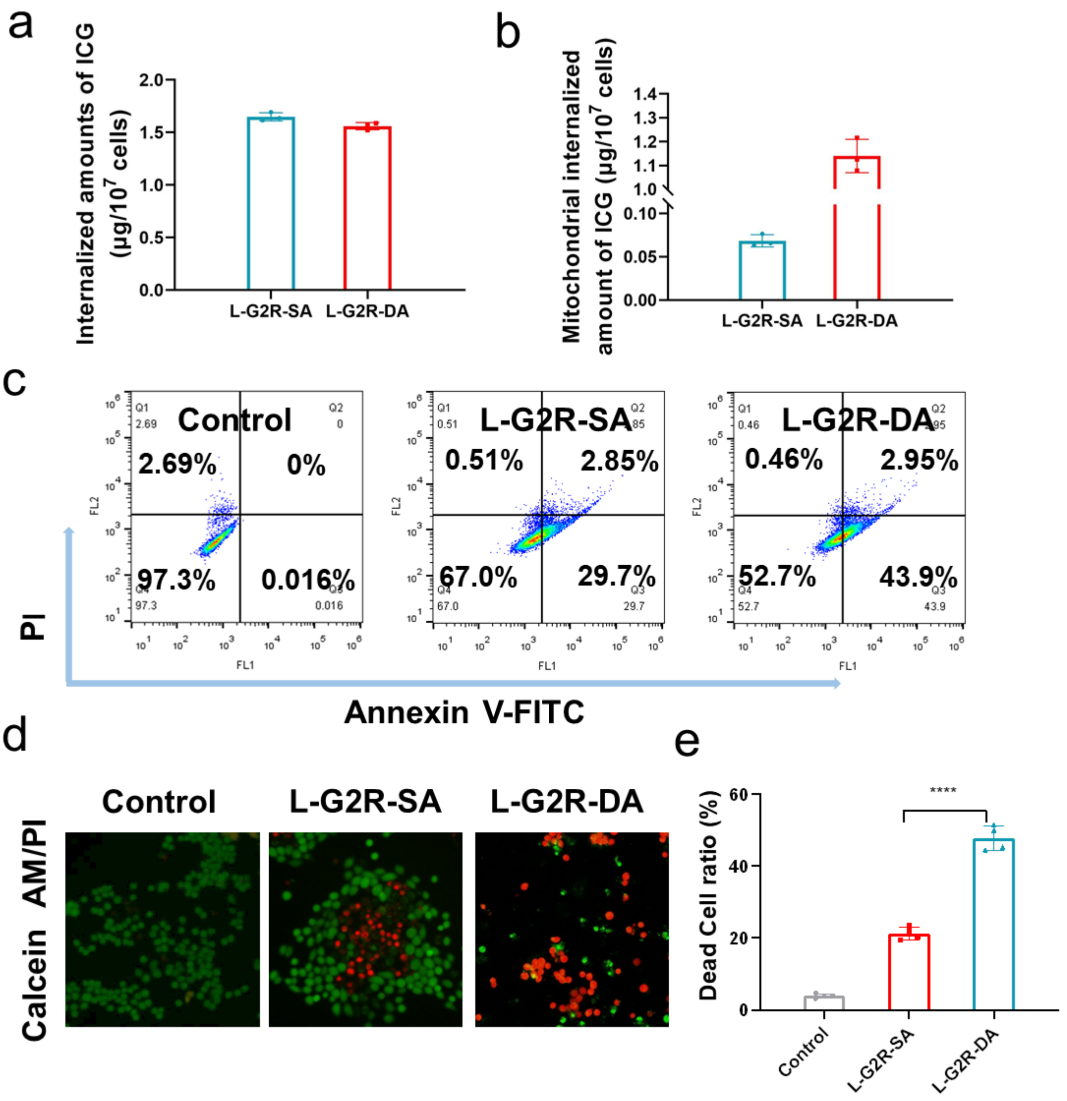


**Supplementary Figure 22**. **a** and **b** Fluorescent spectrometer analysis of ICG accumulation in 4T1 cells and their mitochondria of 4T1 cells for both ICG-loaded liposomes, respectively. Data are presented as means ± SEM. (*n* = 3 biologically independent samples). **c** Flow cytometry analysis of 4T1 cell apoptosis after treated with the culture medium and various ICG containing liposome formulations for 2 h and NIR laser irradiation. **d** Fluorescence microscopic images of 4T1 cells treated with ICG/L-G2R-SA and ICG/L-G2R-DA following laser irradiation. Live/dead cells are green/red (Calcein AM/PI), respectively. **e** Dead cell ratio (cytotoxicity is defined as a number of PI positive cells of the number of the total cells) of 4T1 cells treated with ICG/L-G2R-SA and ICG/L-G2R-DA following laser irradiation. Data are presented as means ± SEM. (*n* = 4 biologically independent samples).

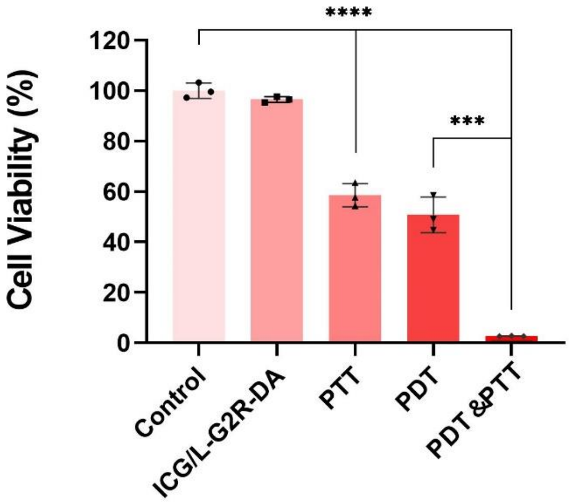


**Supplementary Figure 23.** Relative 4T1 cell viabilities after different treatments (PTT alone, PDT alone, and the combination of PTT and PDT) with ICG/L-G2R-DA (ICG=100 μg/mL) after 24 h of incubation (808 nm laser irradiation, 0.65 W/cm$^2$, 5 min). Data are presented as means ± SEM. ($n$ = 3 biologically independent samples). $P$ values were calculated by the two-tailed Student's $t$-test (***$p = 0.0003$, ****$p < 0.0001$).

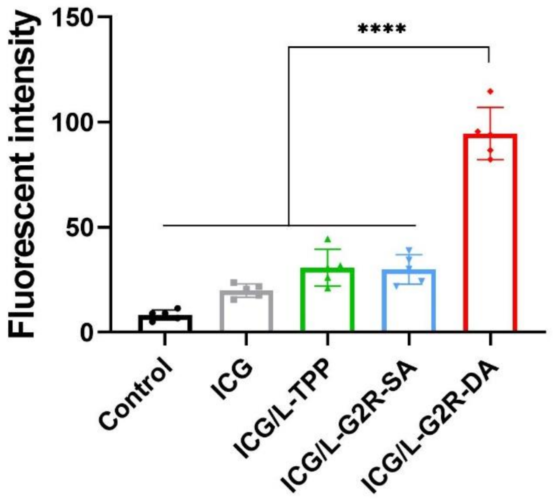


**Supplementary Figure 24**. Semi-quantitative analysis of ROS production test in tumor sections after mice treated with PBS, ICG/L-TPP, ICG/L-G2R-SA, and ICG/L-G2R-DA under NIR laser irradiation. Green fluorescence from ROS stained with $H_2DCFDA$. Data are presented as means ± SEM. (*n* = 5 biologically independent samples). *P* values were calculated by the two-tailed Student's *t*-test (*****p* < 0.0001).

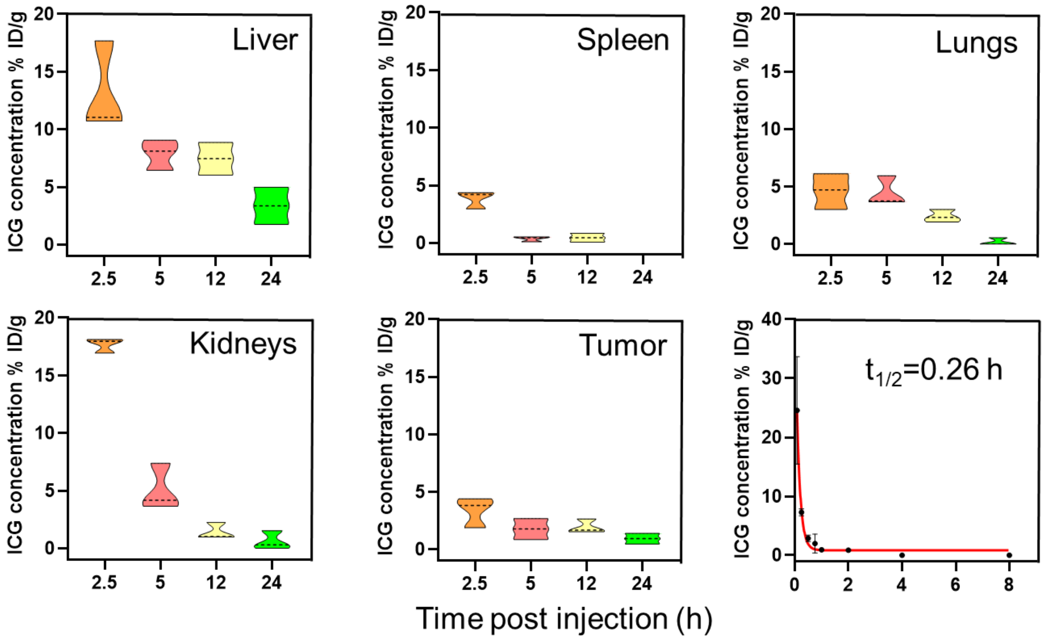


**Supplementary Figure 25.** The bio-distribution profiles of free ICG. Mice bearing 4T1 tumor were injected intravenously with free ICG before sacrificed at different time points. Liver, spleen, lungs, kidneys, tumor and blood sample were collected to measure the concentration of ICG. The violin plot outlines illustrate kernel probablity density, with the area revealing the population of located data. Data are presented as means ± SEM. ($n$ = 3 biologically independent samples).

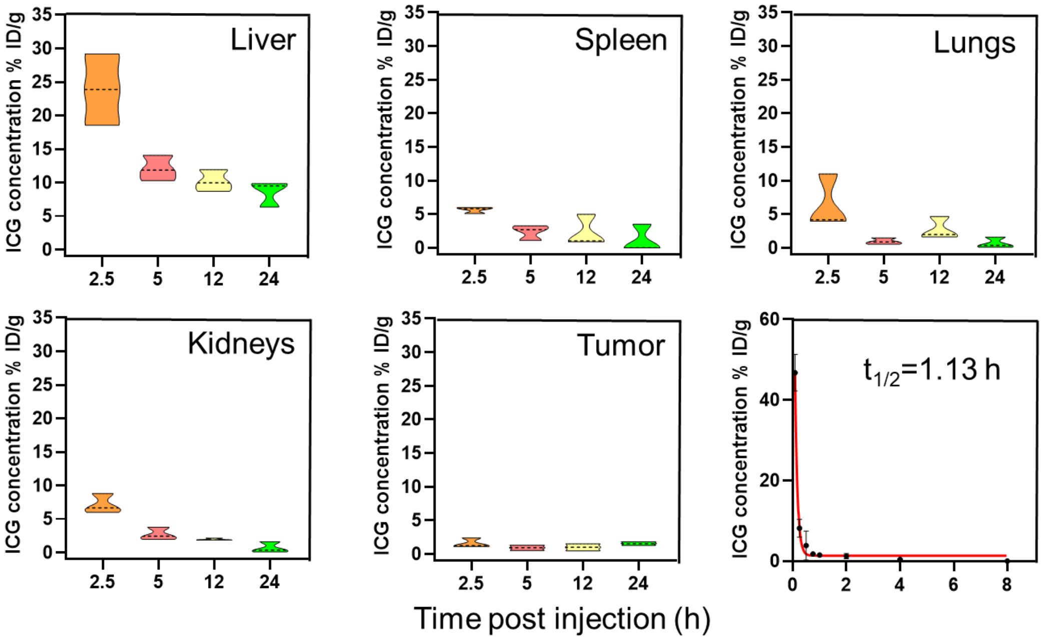


**Supplementary Figure 26.** The bio-distribution profiles of ICG/L-TPP. Mice bearing 4T1 tumor were injected intravenously with ICG/L-TPP before sacrificed at different time points. Liver, spleen, lungs, kidneys, tumor and blood sample were collected to measure the concentration of ICG. The violin plot outlines illustrate kernel probablity density, with the area revealing the population of located data. Data are presented as means ± SEM. (*n* = 3 biologically independent samples).

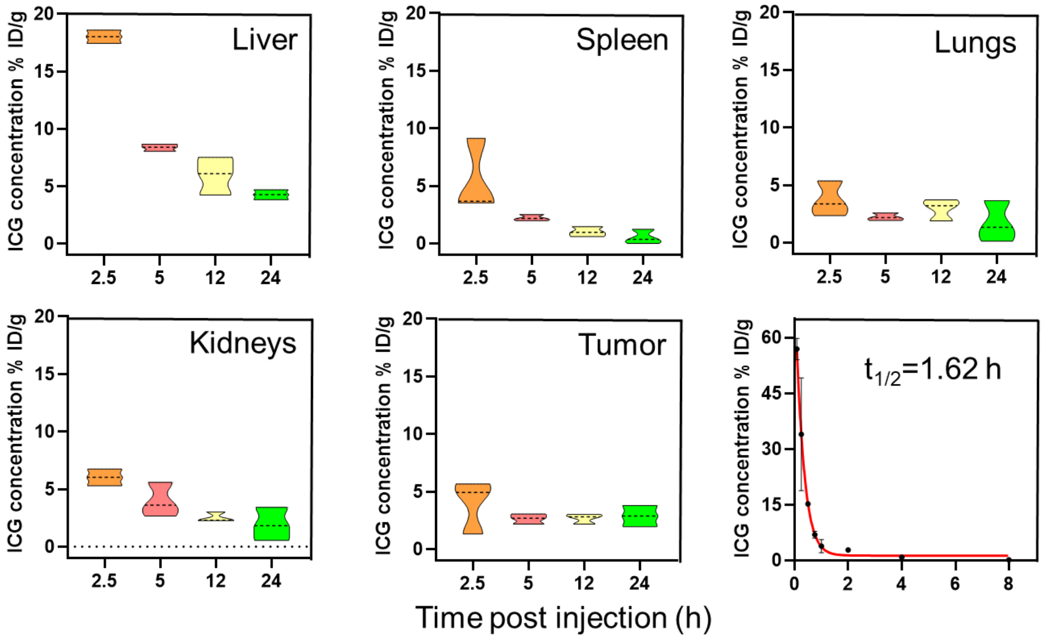


**Supplementary Figure 27.** The bio-distribution profiles of ICG/L-G2R-SA. Mice bearing 4T1 tumor were injected intravenously with ICG/L-G2R-SA before sacrificed at different time points. Liver, spleen, lungs, kidneys, tumor and blood sample were collected to measure the concentration of ICG. The violin plot outlines illustrate kernel probablity density, with the area revealing the population of located data. Data are presented as means ± SEM. (*n* = 3 biologically independent samples).

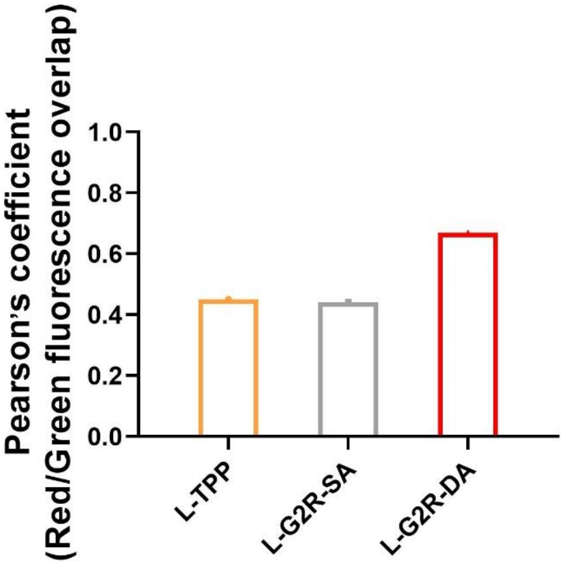


**Supplementary Figure 28**. A Pearson's coefficient was calculated on the basis of the degree of co-localization between the red fluorescent and the green fluorescent from the fluorescence images of Fig 7b.

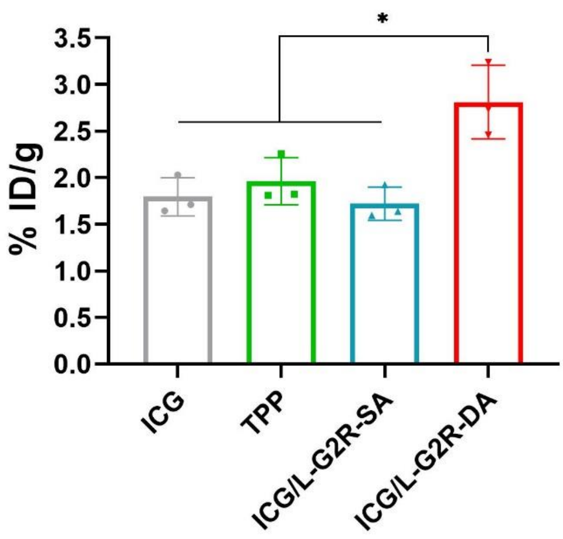


**Supplementary Figure 29**. ICG content in mitochondrial of isolated tumor. Data are presented as means ± SEM. ($n$ = 3 biologically independent samples). $P$ values were calculated by the two-tailed Student's $t$-test. (*$p$ =0.0168).

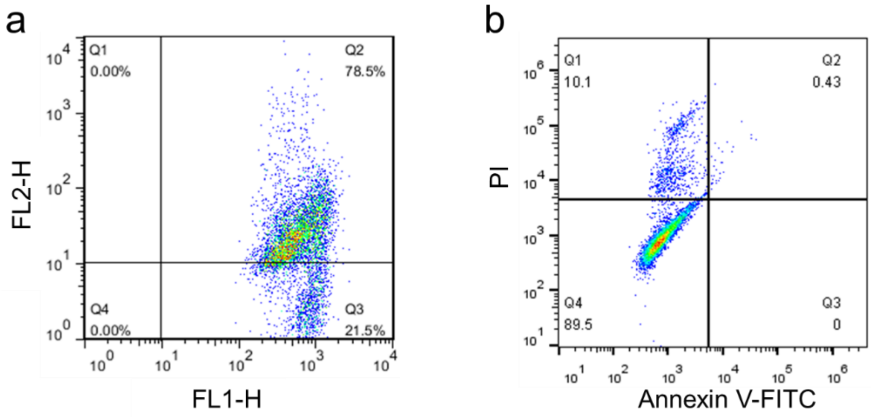


**Supplementary Figure 30.** Gating strategies used for cell sorting. **a** Mitochondrial membrane potential (ΔΨm) and **b** cell apoptosis.
